# Donor Bone Marrow-Derived T Cells Inhibit GVHD Induced by Donor Lymphocyte Infusion in Established Mixed Allogeneic Hematopoietic Chimeras

**DOI:** 10.1371/journal.pone.0047120

**Published:** 2012-10-15

**Authors:** Hui Wang, Yanping Yang, Guanjun Wang, Shumei Wang, Beow Yong Yeap, Megan Sykes, Yong-Guang Yang

**Affiliations:** 1 Columbia Center for Translational Immunology, Columbia University College of Physicians and Surgeons, New York, New York, United States of America; 2 Transplantation Biology Research Center, Massachusetts General Hospital, Harvard Medical School, Boston, Massachusetts, United States of America; 3 First Bethune Hospital of Jilin University, Changchun, Jilin, China; 4 Department of Medicine, Massachusetts General Hospital, Harvard Medical School, Boston, Massachusetts, United States of America; University Paris Sud, France

## Abstract

Delayed administration of donor lymphocyte infusion (DLI) to established mixed chimeras has been shown to achieve anti-tumor responses without graft-vs.-host disease (GVHD). Herein we show that de novo donor BM-derived T cells that are tolerant of the recipients are important in preventing GVHD in mixed chimeras receiving delayed DLI. Mixed chimeras lacking donor BM-derived T cells developed significantly more severe GVHD than those with donor BM-derived T cells after DLI, even though both groups had comparable levels of total T cells at the time of DLI. Post-DLI depletion of donor BM-derived T cells in mixed chimeras, as late as 20 days after DLI, also provoked severe GVHD. Although both CD4 and CD8 T cells contributed to the protection, the latter were significantly more effective, suggesting that inhibition of GVHD was not mainly mediated by CD4 regulatory T cells. The lack of donor BM-derived T cells was associated with markedly increased accumulation of DLI-derived alloreactive T cells in parenchymal GVHD target tissues. Thus, donor BM-derived T cells are an important factor in determining the risk of GVHD and therefore, offer a potential therapeutic target for preventing and ameliorating GVHD in the setting of delayed DLI in established mixed chimeras.

## Introduction

Allogeneic hematopoietic cell transplantation (allo-HCT) remains a potentially curative treatment for leukemias and lymphomas, but its clinical utility has been limited by morbidity and mortality from graft-vs.-host disease (GVHD). Thus, the development of strategies to achieve anti-tumor responses without GVHD has been a major goal in the field of allo-HCT. Donor lymphocyte infusion (DLI), at doses that would induce lethal GVHD in freshly-irradiated mice, mediates effective anti-tumor responses without severe GVHD in established mixed hematopoietic chimeras (MCs) [1]–[3]. The lack of conditioning-induced inflammation at the time of DLI has been shown to be an important factor that prevents trafficking of alloreactive DLI T cells into the epithelial GVHD target tissues in established MCs [4]. Delayed DLI following the establishment of mixed chimerism has also been shown to have the potential to cure hematopoietic malignancies in clinical trials [5]–[7]. However, in comparison to mouse studies in which anti-tumor effects can be reliably achieved by delayed DLI without severe GVHD [1]–[3], a higher incidence of GVHD was noted in mixed chimeric patients after DLI [5]–[7].

In contrast to patients in whom lymphopenia persisted for many months after conditioning, lymphocytes recovered to normal levels quickly in mice after allo-HCT for the establishment of mixed chimerism. It has been shown that T cell depletion immediately before DLI augments GVHD [8], [9]. It was recently found that established lymphocyte-deficient MCs develop GVHD after DLI, whereas those without lymphopenia do not, indicating that lymphopenia at the time of DLI also promotes GVHD in MCs (Li, H. et al, manuscript submitted). In the present study, we assessed the role of donor bone marrow (BM)-derived T cells in the development of GVHD in established MCs after DLI. Our data indicate that donor BM-derived T cells, particularly CD8 T cells that develop de novo in MCs are highly protective against GVHD, and that depletion of these T cells, either prior to or after DLI, significantly augments GVHD regardless of whether or not lymphopenia is present at the time of DLI.

## Materials and Methods

### Animals

Animals were used under protocols approved by the Subcommittee on Research Animal Care of the Massachusetts General Hospital and Columbia University Medical Center. Female wild-type (WT), Rag2^tm1Cgn^/J (RagKO), B6.129S2-Cd4^tm1Mak^/J (CD4KO), and B6.129S2-Cd8a^tm1Mak^/J (CD8KO) mice on the C57BL/6 (B6) background (H-2^b^; CD45.2; Thy1.2); and B6.PL-Thy1a/cy (H-2^b^; CD45.2; Thy1.1) and BALB/c (H-2^d^; CD45.2; Thy1.2) mice were purchased from The Jackson Laboratory (Bar Harbor, Maine). B6-LY5.2/Cr (H-2^b^; CD45.1; Thy1.2) mice were purchased from Frederick Cancer Research Facility (National Institutes of Health, Frederick, MD). Mice were used in experiments at 8 to 12 weeks of age and housed in a specific pathogen-free microisolator environment.

**Figure 1:**
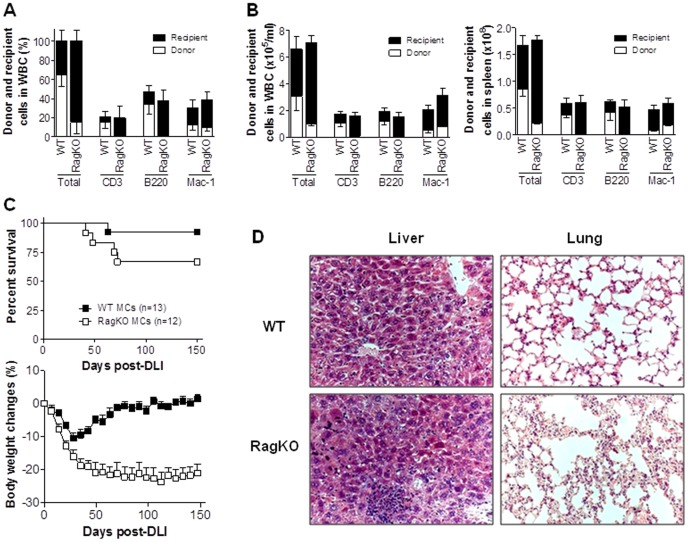
Donor BM-derived lymphocytes attenuate GVHD in mixed chimeras after delayed DLI. Lethally (8 Gy) irradiated BALB/c mice were reconstituted with a mixture of TCD BALB/c plus WT (WT MCs) or RagKO (RagKO BMCs) B6 BMCs 8 weeks before DLI (i.e., injection of 1.5×10^7^ splenocytes) from allogeneic B6 donors. (A). Levels (%) of hematopoietic chimerism in peripheral blood measured 1 week prior to DLI. Data combined from four independent experiments are combined (WT, n = 52; KO n = 47). (B). Absolute donor and recipient cell counts in WBCs (left) and spleen (right) from WT and RagKO at week 1 post-HCT (n = 3 per group). (C). Survivals (top) and body weight changes (bottom) of WT and RagKO MCs. Combined results from 2 independent experiments are shown. (D). Histological analysis (H&E; ×200) of liver and lung tissues from representative WT and RagKO MCs at 150 days after DLI (D).

### Preparation of Mixed Allogeneic Chimeras and Administration of DLI

Mixed chimeras (MCs) were prepared by injection of a mixture of 0.5×10^7^ T cell-depleted (TCD) syngeneic BALB/c and 1.5×10^7^ TCD allogeneic WT, RagKO, CD4KO, or CD8KO B6 BM cells (BMCs) into lethally irradiated (8 Gy) BALB/c mice. TCD BMCs were prepared by depleting CD4^+^ and CD8^+^ cells with anti-CD4 (L3T4) and CD8α (Ly-2) microbeads using the magnetic-activated cell sorter separation system (Miltenyi Biotec, Auburn, CA). T-cell depletion was analyzed by flow cytometry and completeness of depletion (<0.3% cells of the depleted phenotype remaining) was verified in each experiment. DLI was performed using spleen cells (1.5×) from WT B6, B6-LY5.2/Cr (CD45.1) or B6.PL-*Thy1^a^* (Thy1.1) donors 8 weeks after initial TCD BMC injection. Animals were randomized between cages to avoid cage-related bias. Levels of donor chimerism in WBCs were followed up by flow cytometry before and after DLI, in which FITC-conjugated anti-H-2D^d^ mAb 34-2-12 or anti-H-2D^b^ mAb KH95 (BD Biosciences San Diego, CA) was used to distinguish host and donor cells, and in some experiments anti-CD45.1 mAb (A20) and anti-Thy1.1 mAb were used to distinguish between DLI- and BM-derived cells. In vivo depletion of donor BM-derived (Thy1.2^+^) T cells in established MCs was achieved by 4 injections (i.p.) of anti-Thy1.2 mAb (clone 30-H12; the American Type Culture Collection, Manassas, VA) with a 5-day interval starting on day 10 or day 20 after DLI from B6.PL-*Thy1^a^* (Thy1.1) donors.

**Table 1:** Kinetics of bone marrow- and DLI-derived T cells in peripheral blood after DLI.

Wks Post-DLI	Total-T			Total BM-T (Recipient BM-T)			Donor DLI-T		
	WT	RagKO	*p*	WT	RagKO	*p*	WT	RagKO	*p*
0	1.72±0.26	1.55±0.14	0.094	1.72±0.26 (1.05±0.21)	1.55±0.14	0.094	N/A	N/A	N/A
2	0.55±0.14	0.47±0.02	0.181	0.50±0.14 (0.10±0.02)	0.10±0.01	0.015	0.05±0.01	0.37±0.02	0.002
4	1.18±0.02	0.26±0.01	0.0004	1.01±0.02 (0.01±0.005)	0.02±0.002	0.001	0.17±0.01	0.24±0.01	0.04

Numbers are absolute cell counts of total T cells (Total-T), BM-derived T cells (BM-T) and DLI-derived T cells (DLI-T) in the WBCs (mean±SDs; ×10^5^/mL) at the indicated time points.

**Figure 2:**
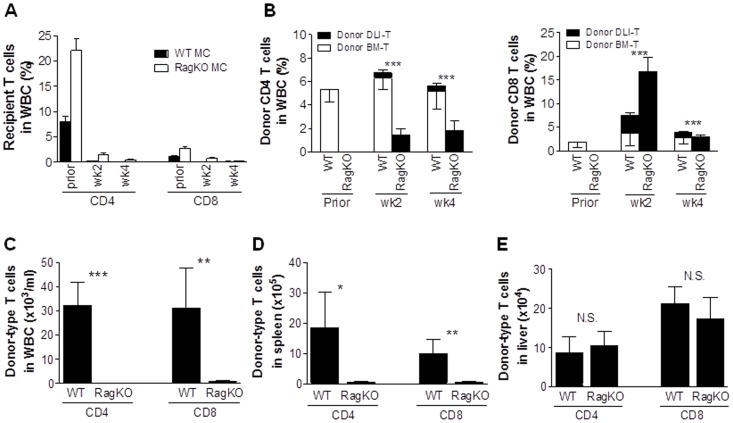
T cell reconstitution in mixed chimeras after delayed DLI. MCs were prepared by injection of a mixture of TCD BALB/c plus WT (CD45.2^+^) or RagKO (CD45.2^+^) B6 BMCs into lethally-irradiated BALB/c mice, and injected 8 weeks with 1.5×10^7^ of splenocytes from B6-LY5.2/Cr (CD45.1^+^) donors. (A–B) Levels of recipient (A) and donor BM (CD45.2^+^KH95^+^)- and DLI (CD45.1^+^KH95^+^)-derived CD4 and CD8 T cells (B) in WBCs of WT and RagKO MCs at the indicated times (7–9 mice per group were analyzed at each time point). Prior denotes 1 week prior to DLI. ***, p<0.005 for comparison of donor DLI-derived T cells (▪) between WT and RagKO MCs at the indicated time points. (C–E) Levels of donor T cells in WBCs (C), spleen (D) and liver (E) from WT (n = 3) and RagKO (n = 3) MCs measured at the end (150 days post-DLI) of the experiment. Values shown are mean ± SEM. N.S., not significant; *, p<0.05; **, p<0.01; ***, p<0.005 for comparison between WT and RagKO MCs.

### Histologic Analysis

Carcasses were saved in 10% formalin after animals were sacrificed for autopsy. Tissues (liver, lung and intestine) were embedded in paraffin, sectioned, and stained with hematoxylin and eosin. Slides were observed using an Olympus BX40 light microscope (Olympus, Melville, NY) with 20×/0.5 numeric aperture (NA) objectives, and photographed using a Hitachi HV-C20 color camera (Hitachi, Nashua, NH).

**Table 2:** Accumulation of donor DLI-derived T cells in parenchymal, but not lymphoid, tissues in RagKO chimeras (Mean±SDs).

T-cell Origin	WBC (x10^3^/ml)			Spleen (x10^5^)			Liver (x10^4^)		
	WT	RagKO	*p*	WT	RagKO	*p*	WT	RagKO	*p*
CD4									
Donor DLI	0.01±0.01	0.33±0.06	0.187	0.22±0.06	0.57±0.37	0.096	0.49±0.4	10.2±6.50	0.029
Donor BM	32.1±17.1	N/A	N/A	18.37±20.09	N/A	N/A	8.07±7.11	N/A	N/A
CD8									
Donor DLI	0.34±0.57	0.35±0.6	0.492	0.19±0.11	0.5±0.44	0.166	0.75±0.42	17.3±9.26	0.018
Donor BM	37±20.4	N/A	N/A	9.73±8.12	N/A	N/A	20.47±7.37	N/A	N/A

N/A, not available.

**Figure 3:**
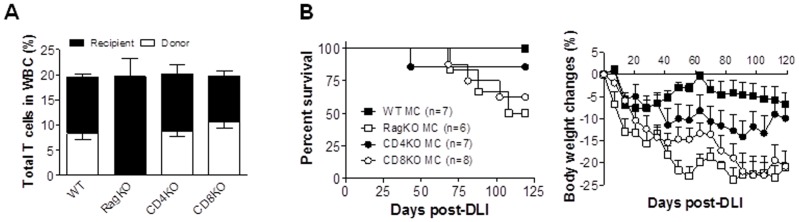
Both donor BM-derived CD4 and CD8 T cells are protective against GVHD in mixed chimeras receiving delayed DLI. Lethally (8 Gy) irradiated BALB/c mice were reconstituted with a mixture of TCD BALB/c plus WT (WT MC; n = 7), RagKO (RagKO MC; n = 6), CD4KO (CD4KO MC; n = 7), or CD8KO (CD8KO MC; n = 8) B6 BMCs 8 weeks before DLI from WT B6 donors. (A) Hematopoietic chimerism in WBCs measI can hear Kazured one week prior to DLI. (B) Survival (left) and body weight changes (right).

**Table 3:** T cell reconstitution in peripheral blood prior to DLI.

Group	Total CD4	*p* *	Total CD8	*p* *
WT MC	16.9±3.1		2.5±1.5	
RagKO MC	17.7±7.6	0.417	2.0±1.2	0.250
CD4KO MC	9.7±5.0	0.004	10.4±2.8	<0.001
CD8KO MC	19.0±5.2	0.178	0.8±0.2	0.010

Data are presented as percentages of the indicated cell populations (mean±SDs) in WBCs.

* depicts the p value for comparison between the indicated group and WT MCs; N/A, not available.

### Statistical Analysis

Statistical analysis of survival data was performed with the log rank test. Student’s *t* test was used to determine the level of significance of differences in group means. Body weight was transformed by taking the square root prior to fitting the trend over time using a mixed linear model with random intercepts and slopes assumed for each mouse. A quadratic effect was included in the models to allow for initial weight loss followed by subsequent gain. An unstructured covariance matrix was specified for the random effects, while a first-order autoregressive [AR(1)] error structure was assumed for the repeated measurements within animals. As the treatment groups were assigned randomly, an overall mean was assumed for the population of mouse-specific intercepts. For the analysis focusing on the weight differences starting at week 5, the model incorporated separate fixed effects for the group means in place of an overall mean intercept. Comparison between experimental groups was based on hypothesis tests of the fixed-effects contrasts between specified groups with inference based on the *F*-test. Estimation by restricted maximum likelihood (REML) was computed using SAS 9.2 (Cary, NC). A p value of <0.05 was considered to be significant in both types of analysis.

**Figure 4:**
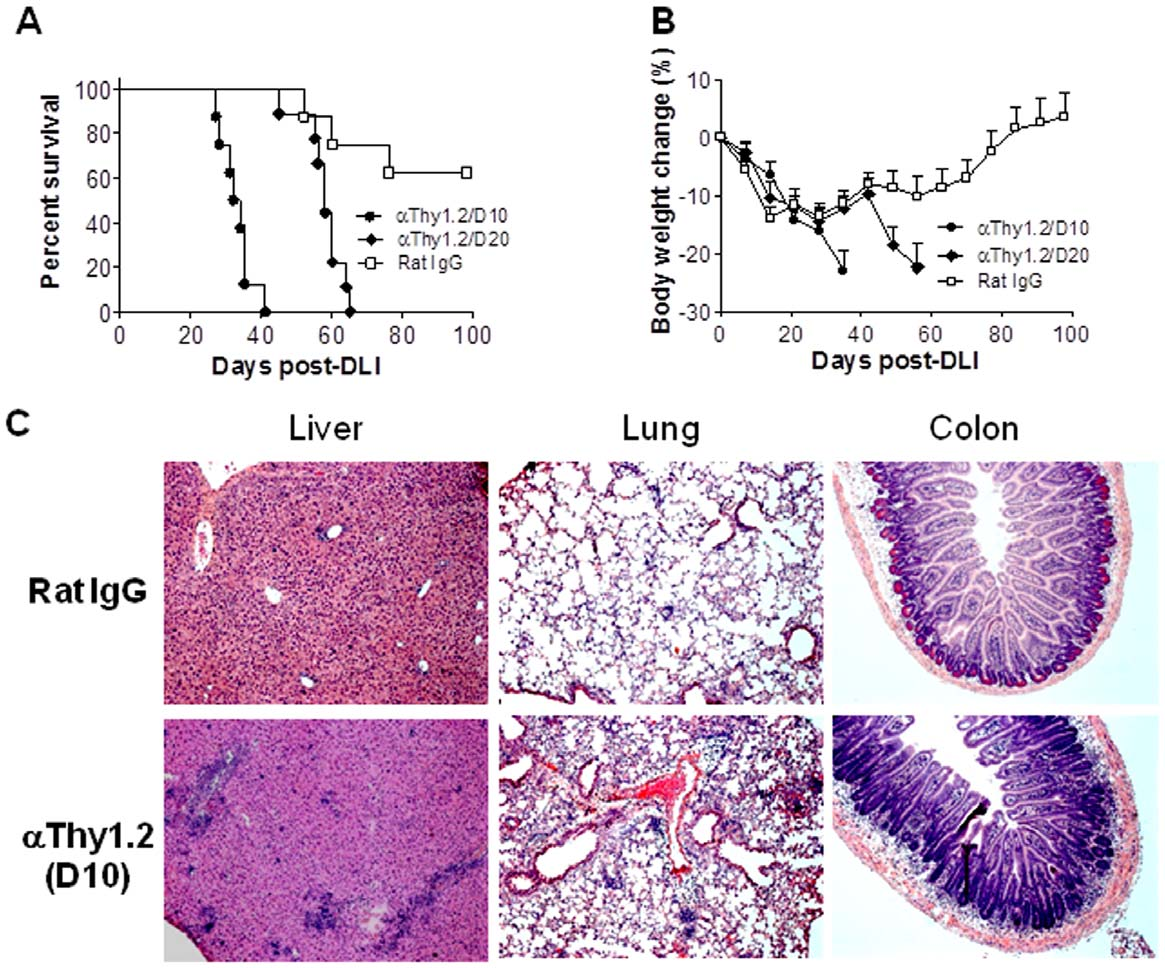
Post-DLI depletion of donor BM-derived T cells exacerbates GVHD in mixed chimeras. Lethally (8 Gy) irradiated BALB/c mice were reconstituted with a mixture of TCD BALB/c plus WT B6 (Thy1.2+) BMCs 8 weeks before DLI (i.e., injection of 1.5×10^7^ splenocytes) from B6.PL-Thy1a (Thy1.1+) donors. Donor BM-derived T cell depletion in these MCs was performed by injection (i.p.) of anti-Thy1.2 antibodies starting at day 10 (n = 8) or day 20 (n = 9) after DLI as described in Materials and Methods. Control MCs (n = 8) were treated with Rat IgG. Shown are survival (A), body weight changes (B) and histological analysis (H&E; ×100) of liver, lung and colon from representative MCs at day 20-post antibody treatment (i.e., 30 days post-DLI) (C).

**Figure 5:**
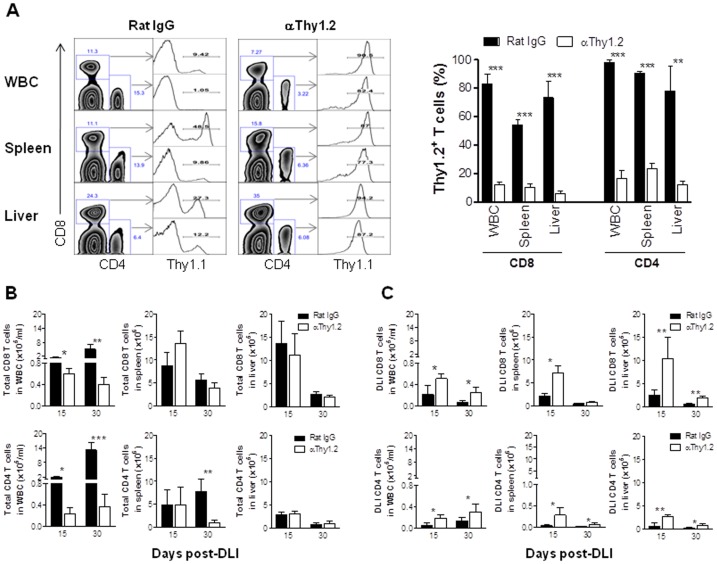
Donor DLI-derived T cell expansion following the depletion of donor BM-derived T cells. MC preparation and DLI administration were performed as detailed in the legend to Figure 4, and treated with anti-Thy1.2 or Rat IgG starting at day 10 post-DLI. MCs were sacrificed 5 (n = 3 per group) and 20 (Rat IgG, n = 3; αThy1.2, n = 4) days after antibody treatment (i.e., 15 and 30 days after DLI, respectively), and the levels of Th1.1+ and Thy1.2+ T cells in various tissues were determined by flow cytometry using anti-Thy1.1 mAb. (A) Representative staining profiles (Left) and levels (mean±SEM) of Thy1.2^+^ (i.e., cells stained negative for Thy1.1) T cells in various tissues at day 5 post-antibody treatment (15 day after DLI). (B,C) Numbers (mean±SEM) of total (B) and DLI-derived Thy1.1+ (C) T cells (CD8 and CD4 T cells are shown at the top and bottom panels, respectively) in WBC, spleen and liver at the indicated time points. *, p<0.05; **, p<0.01; ***, p<0.005.

## Results

### Lack of Donor BM-derived Lymphocytes Provokes GVHD in Established Fully MHC-mismatched Mixed Chimeras Receiving Delayed DLI

MCs were prepared by injecting mixed TCD BMCs from BALB/c plus WT B6 or RagKO B6 mice into lethally-irradiated BALB/c mice. Flow cytometric analysis at week 7 post-BM transplantation (BMT) revealed that multilineage mixed chimerism was established in the MCs that received WT B6 BMCs, while the MCs receiving RagKO BMCs showed split mixed chimerism with myeloid (e.g., Mac-1^+^) cells of both recipient and donor origin, but lymphocytes (i.e., CD3^+^ T and B220^+^ B cells) only of recipient origin (Figure 1A–B). However, the two groups of MCs showed similar levels of total CD3^+^, B220^+^ and Mac-1^+^ cells (Figure 1A–B).

To determine the role of donor BM-derived lymphocytes in DLI-mediated GVHD, WT and RagKO MCs were given DLI from WT B6 donors at week 8 post-BMT, and followed for body weight changes, survival rates and histological GVHD. Although only a small difference was seen in survival rates between WT (92.3%) and RagKO (66.7%) MCs (Figure 1C), the latter MCs showed significantly more severe body weight loss than the former group (Figure 1C; p<0.001). WT MCs exhibited moderate body weight loss early after DLI followed by recovery, whereas RagKO MCs showed continued weight loss throughout the experiment (Figure 1C). Histological examination confirmed severe inflammatory infiltrates and the associated tissue lesions in GVHD target organs (i.e., lung and liver) from the long-term surviving RagKO, but not WT, MCs (Figure 1D). These results indicate that the lack of pre-existing donor BM-derived lymphocytes is an important risk factor for persistent GVH responses and GVHD in established MCs after delayed DLI.

### Increased Expansion and Survival of Donor DLI-derived Allogeneic T Cells in RagKO Mixed Chimeras

We also assessed the kinetics of recipient and donor T cells in peripheral blood of WT vs. RagKO MCs after DLI. In order to distinguish between donor BM- and DLI-derived T cells, MCs were prepared by injecting mixed TCD BMCs from BALB/c plus WT (CD45.2^+^) or RagKO (CD45.2^+^) B6 mice into lethally-irradiated BALB/c mice, and injected 8 weeks later with splenocytes from congeneic B6-LY5.2/Cr (CD45.1^+^) donors. In both WT and RagKO MCs, recipient type (BALB/c) T cells were rapidly eliminated within 2 weeks after DLI (Figure 2A and Table 1). Compared to WT MCs, in which donor BM-derived T cells remained the major donor T cell population, a significantly greater expansion of DLI-derived donor (CD45.1^+^KH95^+^) T cells was seen in RagKO MCs at weeks 2 and 4 post-DLI (Figure 2B and Table 1). The fact that lack of donor BM-derived lymphocytes was associated with more severe GVHD and greater expansion of DLI-derived T cells in RagKO MCs (and vice versa in WT MCs) suggests that donor BM-derived non-alloreactive T cells in established MCs may suppress the alloresponses of DLI T cells.

We also measured T cell chimerism in long-term surviving MC recipients that were sacrificed at day 150 post-DLI. All these long-term surviving WT and RagKO MCs became full donor chimeras (data not shown), indicating complete elimination of recipient hematopoietic cells by DLI-induced alloresponses. DLI-derived donor T cells were barely detected in peripheral blood or spleen from both WT and RagKO MCs, while WT MCs demonstrated good reconstitution with donor BM-derived T cells in these tissues (Table 2 and Figure 2C,D). Surprisingly, despite the extremely poor T cell repopulation in lymphoid tissues in RagKO MCs, the levels of donor T cells in the liver of these mice were comparable to those of WT MCs (Figure 2E). However, unlike the WT MCs, in which almost all T cells were donor BM-derived, T cells in the liver of RagKO MCs were all DLI-derived (Table 2) and presumably long-term surviving alloreactive T cells. The data correlated well with the pathological findings (Figure 1D) in RagKO MCs.

### Both Donor BM-derived CD4 and CD8 T Cells Mediate Protection Against GVHD Induced by DLI in Established Mixed Chimeras

The potential role of donor BM-derived CD4 and CD8 T cells in regulation of DLI T cell alloresponses was assessed by comparing GVHD development among WT MCs, RagKO MCs, and MCs that were prepared by injection of syngeneic plus CD4KO (CD4KO MCs) or CD8KO (CD8KO MCs) allogeneic BMCs. Although significant differences were detected in the levels of CD4 and CD8 T cell subsets (Table 3), the overall T cell levels were comparable among these MCs prior to DLI (Figure 3A). Consistent with the results in Figure 1, DLI given at week 8 induced significantly more severe GVHD in RagKO MCs than in WT MCs (Figure 3B). Although CD4KO MCs showed more profound body weight loss starting at 5 weeks than WT MCs (p<0.01), these MCs had less severe GVHD, as shown by lower mortality and significantly improved body weight recovery than CD8KO (p<0.001 starting at 5 weeks after DLI) and RagKO (p<0.05 for the entire period of observation) MCs (Figure 3B). The survival rates and body weight changes were, in general, comparable between CD8KO and RagKO MCs, with the exception that the latter group showed significantly more severe body weight loss starting at 5 weeks after DLI (p<0.05). These results indicate that both BM-derived CD4 and CD8 T cells mediate protection against DLI-induced GVHD, but the latter cell population is more effective.

### Depletion of Donor BM-derived T Cells in Established Mixed Chimeras after DLI Provokes GVHD

Although lymphopenia at the time of DLI may potentially promote GVHD [8], RagKO MCs did not show lymphopenia compared to WT MCs at the time of DLI (Figure 1A–B; Table 1). However, lymphopenia was detected at the later times in RagKO MCs when recipient BM-derived cells were eliminated (Table 1; Figure 2), suggesting that the continuous presence of donor BM-derived T cells might be required to inhibit GVHD. To address this question, we assessed the effect of post-DLI depletion of donor BM-derived T cells on GVHD. WT MCs were prepared by injection of BALB/c and Thy1.2^+^ B6 mouse BMCs into lethally-irradiated BALB/c mice (Thy1.2^+^). These MCs received DLI 8 weeks later from B6.PL-Thy1a (Thy1.1^+^) donors. We treated these MCs with anti-Thy1.2 mAb to deplete BM-derived T cells (or with Rat IgG as the control) starting at day 10 or day 20 after DLI, when recipient-type T cells were almost completely eliminated (Table 1; Figure 2A). MCs treated with anti-Thy1.2 mAb starting at either 10 or 20 days after DLI developed significantly more severe GVHD than rat IgG-treated controls (Figure 4). Anti-Thy1.2 mAb-treated MCs showed significantly increased mortality (Figure 4A), as well as more severe body weight loss (p<0.05; Figure 4B), hunched posture, and diarrhea before death compared to rat IgG-treated control MCs. Furthermore, histological analysis revealed more severe inflammatory infiltrates and tissue lesions in GVHD target organs (e.g., liver, lung and colon) from MCs that were treated with anti-Thy1.2 mAb compared to those from rat IgG-treated controls (Figure 4C).

Some MCs that were treated with anti-Thy1.2 or rat IgG starting at day 10 post-DLI were sacrificed 5 and 20 days after first antibody injection (i.e., 15 and 30 days after DLI), and DLI-derived donor T cell expansion in peripheral blood, spleen and liver was measured by flow cytometric analysis. The levels of Thy1.2^+^ CD4 and CD8 T cells in MCs treated with anti-Thy1.2 were significantly reduced compared to rat IgG-treated controls, reflecting an efficient T cell depletion by anti-Thy1.2 mAb (Figure 5A). With the exception of peripheral blood, depletion of donor BM-derived T cells did not result in a significant reduction in total CD8 T cell levels in these tissues (Figure 5B). Anti-Thy1.2- and rat IgG-treated MCs also showed comparable levels of total CD4 T cells in spleen at day 15 post-DLI and in liver at days 15 and 30 post-DLI (Figure 5B). However, depletion of donor BM-derived Thy1.2^+^ T cells led to significantly greater expansion and/or survival of DLI-derived Thy1.1^+^ T cells in all tissues examined (Figure 5C). Taken together, these results indicate that the continuous presence of donor BM-derived T cells is essential for preventing GVHD in established MCs after DLI.

## Discussion

The present study provides evidence that donor BM-derived T cells, particularly CD8 T cells that develop post-BMT in the presence of recipient antigens, are highly protective against GVHD in established MCs receiving delayed DLI. DLI induced significantly more severe GVHD in RagKO MCs that had no donor BM-derived T cells at the time of DLI or in WT MCs that were depleted of donor BM-derived T cells after DLI compared to untreated WT MCs. The lack of donor BM-derived T cells was associated with markedly increased expansion and infiltration into GVHD target tissues of DLI-derived alloreactive T cells. Lymphopenia has the potential to promote anti-tumor responses of adoptively transferred T cells by increasing expansion of T cells with associated development of effector function [10], decreasing capacity for regulation of immune responses by Tregs [11], [12], and failing to prevent microbial translocation-induced inflammatory stimuli [13]. Total T cell depletion immediately before allo-HCT has been shown to promote GVHD both in human and murine models [9], [14]. However, the increased risk for GVHD observed in the established RagKO MCs following DLI in the present study was not caused by lack of T cells at the time of DLI, because RagKO MCs did not show reduced T cell counts compared to WT MCs at the time of DLI (Figure 1 and Table 1) and depletion of donor BM-derived T cells 20 days after DLI also significantly exacerbated GVHD in established WT MCs (Figure 4).

DLI-derived T cells were barely detectable in the blood and spleen from long-term surviving MCs (Table 2), regardless of the presence or absence of donor BM-derived T cells. As a consequence of the loss of DLI-derived cells, lymphoid tissues in RagKO, but not WT, MCs were found to be lymphopenic at later times (Figure 2C,D), which was associated with a more severe GVHD and increased accumulation of DLI-derived alloreactive T cells in parenchymal GVHD target tissues. This is consistent with the previous finding that alloreactive T cells have impaired ability to reconstitute host lymphoid organs in allo-HCT recipients [15]–[17]. These results indicate that MCs lacking donor BM-derived T cells will likely develop lymphopenia eventually after DLI when recipient hematopoietic cells are eliminated by GVHR. Such post-DLI lymphopenia developing after the onset of GVHD is likely to exacerbate GVHD by providing an additional stimulus driving continuous expansion of alloreactive effector/memory T cells. Furthermore, persistent lymphopenia may also provoke GVHD by diminishing protection against microbial translocation-induced inflammatory stimuli [13], a potent risk factor for GVHD [4].

Infusion of donor CD4^+^CD25^+^ Tregs, which are critical for maintenance of self-tolerance and prevention of autoimmunity, has been found to effectively inhibit GVHD [18], [19]. Although various types of Tregs have been reported, CD4^+^CD25^+^Foxp3^+^ Tregs are considered the most robust Treg population [20]. In lethally-irradiated full donor chimeras, it was found that depletion of recipient T cells prior to DLI promotes GVHD, and that the exacerbation of GVHD in this model was due to removing CD4^+^CD8^−^ and CD4^−^CD8^−^, but not CD8^+^, Tregs [8], [14]. However, our data indicate that that the protective effect of donor BM-derived T cells against GVHD induced by delayed DLI in established chimeras was not mediated predominantly by CD4^+^ Tregs. Although both CD4 and CD8 T cells were found to be protective against GVHD induced by DLI in established MCs, the development of significantly more severe GVHD in CD8KO MCs than in CD4KO MCs suggests that donor BM-derived CD8 T cells provide more potent protection than donor BM-derived CD4 T cells. It has been reported that CD4 Tregs developing in the absence of CD8 T cells exhibit poor suppressive function in a mouse allergic lung disease model [21]. However, CD4 Tregs in the CD8KO MCs are expected to be functional, as CD8 T cells were present in these MCs both prior to (recipient BM-derived CD8 T cells) and after (recipient BM- and DLI-derived CD8 T cells) DLI. Furthermore, the role for CD8 T cells in CD4 Treg function may not apply to all immunological settings. It has been reported that a treatment that inhibits alloresponses via a Treg-dependent mechanism can also significantly prolong allograft survival in CD8 KO mice [22]. Furthermore, CD8KO BMT in lethally-irradiated mice can lead to reconstitution of functional CD4 Tregs that are capable of suppressing GVHD [8]. Taken together, CD4 Tregs in CD8KO MCs are likely to be functional and therefore, the more severe GVHD in CD8KO MCs than in CD4KO MCs after DLI suggests that the protective effect against GVHD of donor BM-derived T cells is attributed more to donor BM-derived CD8 than CD4 T cells. Although the development and function of CD8^+^Foxp3^+^ Tregs are less understood than CD4 Tregs, such Treg cell populations have been identified in various models [23]–[26]. Unlike CD4^+^Foxp3^+^ Tregs, CD8^+^Foxp3^+^ Treg function was found to be positively regulated by IL-6 [27], a cytokine that has been shown to increase in mice with GVHD [28]. Further studies are needed to determine whether the protective effect against GVHD of donor BM-derived CD8 T cells is attributable to the induction of CD8^+^Foxp3^+^ Tregs.

In summary, the present study demonstrates that donor BM-derived T cells are an important factor determining the risk of GVHD in established MCs receiving delayed DLI. Exacerbation of GVHD was seen not only in MCs having no donor BM-derived T cells at the time of DLI, but also in those losing donor BM-derived T cells a few weeks after DLI. Thus, the level of donor BM-derived T cells in MCs may provide a better outcome predictor than recipient T cells after DLI and allow for optimization of the timing of DLI administration. Furthermore, because donor T cells developing de novo in the recipients are tolerant of both donor and recipient antigens [29], our results indicate that improving T cell development from donor BM cells and infusing allodepleted or non-alloreactive donor T cells may offer an effective means to prevent or inhibit GVHD induced by delayed DLI in established MCs.
